# Ultra-Fine Bubble Distributions in a Plant Factory Observed by Transmission Electron Microscope with a Freeze-Fracture Replica Technique

**DOI:** 10.3390/nano8030152

**Published:** 2018-03-08

**Authors:** Tsutomu Uchida, Hitoshi Nishikawa, Nobuki Sakurai, Masashi Asano, Naoki Noda

**Affiliations:** 1 Faculty of Engineering, Hokkaido University, Sapporo, Hokkaido 060-8628, Japan; 2 Minamata Research Center, JNC Co., Minamata, Kumamoto 867-8501, Japan; h.nishikawa@jnc-corp.co.jp (H.N.); n.sakurai@jnc-corp.co.jp (N.S.); n.noda@jnc-corp.co.jp (N.N.); 3 Business Promotion Office, JNC Co., Chiyoda-ku, Tokyo 100-8105, Japan; m.asano@jnc-corp.co.jp

**Keywords:** ultra-fine bubble (UFB), freeze-fracture replica method, transmission electron microscope observation, plant factory

## Abstract

Water containing ultra-fine bubbles (UFB) may promote plant growth. But, as UFBs are too small to distinguish from other impurities in a nutrient solution, it is not known if UFBs survive transport from the water source to the rhizosphere. Here we use the freeze-fracture replica method and a transmission electron microscope (TEM) to observe UFBs in the nutrient solutions used in a crop-growing system known as a plant factory. In this factory, TEM images taken from various points in the supply line indicate that the concentration of UFBs in the nutrient solution is conserved, starting from their addition to the nutrient solution in the buffer tank, through the peat-moss layer, all the way to the rhizosphere. Measurements also show that a thin film formed on the surface of UFBs in the nutrient solution, with greater film thickness at the rhizosphere. This film is considered to be made from the accumulation of impurities coming from solute and the peat-moss layer.

## 1. Introduction

Recent attention has been given to industrial application of ultra-fine bubbles, or UFBs, which are bubbles of diameter below 1 μm. For example, in agriculture, UFB-added solution has been tested as a growth promoter. In one such test, Ebina et al. [1] showed that the growth of hydroponically cultivated *Brassica campestris* over four weeks was significantly promoted in air-UFB-added water compared to that with normal water. Park & Kurata [2] reported that UFB-added water produced about twice as much lettuce growth over the control case in a lettuce plant factory. Soon thereafter Ushikubo et al. [3] observed that the cytoplasmic streaming rate of coleoptile cells was increased by oxygen (O_2_)-UFB-added water. And more recently, Liu et al. [4] showed that the germination rate of barley seeds was significantly increased by O_2_-UFB-added water. These studies have shown that the growth of plants can be promoted by using UFB-added water. Following these results, we have been running experiments to ascertain whether UFB-added water is effective for promoting the growth of midi tomatoes in a plant factory.

However, there is a knowledge gap between the physical-property studies of UFBs in the laboratory and UFB use in an actual system. In pure water, UFBs are usually observed in the particle tracking analysis method or in a dynamic light scattering method, but as neither method can distinguish between bubbles and impurity particles, UFB observations have not been used in an actual solution system. When UFB-added water is applied to an actual system such as a plant factory, the aqueous solution (or the nutrient solution) used for the plant contains various impurities. As we cannot distinguish the UFBs in such an aqueous solution containing impurities, the existence of UFBs in the plant-factory solution has yet to be confirmed.

The only measurement method applicable to this system is the freeze-fracture replica method applied to the solution [5]. This method involves quenching the solution at liquid-nitrogen temperatures, thus fixing solutes such as UFBs in the ice matrix, and then creating a replica of the cross-sectional surface fractured at a low temperature under vacuum. Then, the replica is observed with a transmission electron microscope (TEM). This method has been used to demonstrate the purification process of wastewater with applied UFBs [6]. This observation also indicated that there is a sufficient amount of UFBs after industrial UFB generation for the method to be useful [7].

In this study, we investigate the distribution of UFBs in the nutrient fluid used in the JNC Corporation’s plant factory in Kumamoto, Japan. Here, midi tomatoes are grown on peat moss, on which the UFB-added solution drips. Unlike hydroponic culture, the nutrient solution of the plant factory is non-circulating, making it more difficult to confirm the existence of UFBs in the supplied solution. To examine the UFBs, we sample the solution from the supply tank of UFB-added nutrient solution (initial condition), from the supply-pipe (transporting condition), and from the puddle near the root after being supplied to the plant body (near-plant condition). The freeze-fracture replica–TEM observations reveal the distribution and state of the UFBs in each solution sample. Then, we confirm that UFBs are sufficiently supplied to the vicinity of the plant roots. Finally, we examine the UFBs size distribution and shape features at the near-plant condition, and discuss how this may affect plant growth.

## 2. Background

Fine bubbles (FB) refer to all bubbles with a diameter *d* below 100 μm [8], and are subdivided into those above 1 μm (micro bubbles, or MBs) and the UFBs below 1 μm. UFBs are more difficult to observe directly because their size is below the spatial resolution of the optical microscope. For example, with a wavelength λ = 600 nm, the scattered light from UFBs smaller than about λ/π, that is, a diameter *d* < 190 nm, becomes very weak.

UFBs have different physical properties than MBs. One of them is that UFBs have much smaller terminal velocities than MBs. Usually, the rising speed *Vs* of a particle in water is defined by the Stokes equation:

*Vs* = *d*^2^ (ρ_p_ − ρ_f_) g/18μ, (1)

where ρ_p_ and ρ_f_ are densities of particle and surrounding liquid, g is the gravitational acceleration, and μ is the liquid viscosity. This equation shows *Vs* decreases as the square of the decrease in particle diameter. As a result, one can observe that MB-added water changes from milky to transparent after several minutes of bubble generation by the rising and disappearance of MBs. However, it is thought that UFBs will stay in the liquid phase for a long time with their small rising rates. Furthermore, since UFBs have negative ζ-potentials of several tens of mV [9], UFBs are considered to be inhibited from their coalescence. Thus, *Vs* for UFB does not increase due to an increase in *d*.

Another special property of UFBs is their larger internal pressure. The bubble internal-pressure ∆*P* is given by the Young-Laplace equation:

∆*P* = 4σ/*d*, (2)

where σ represents the air-liquid interfacial tension. This equation shows that ∆*P* increases rapidly as *d* decreases. As gas dissolves in water according to Henry’s law, UFBs are thought to shrink faster than MBs due to their higher internal pressure.

These two contradictory properties in the lifetime of UFBs in water lead a paradox about the stability of UFB (Epstein-Plaset theory [10]). Moreover, as UFB cannot be observed with an optical microscope easily, the existence of UFBs has been debated [11].

## 3. Results and Discussion

### 3.1. TEM Observations on Freeze-Fracture Replicas

**Figure 1:**
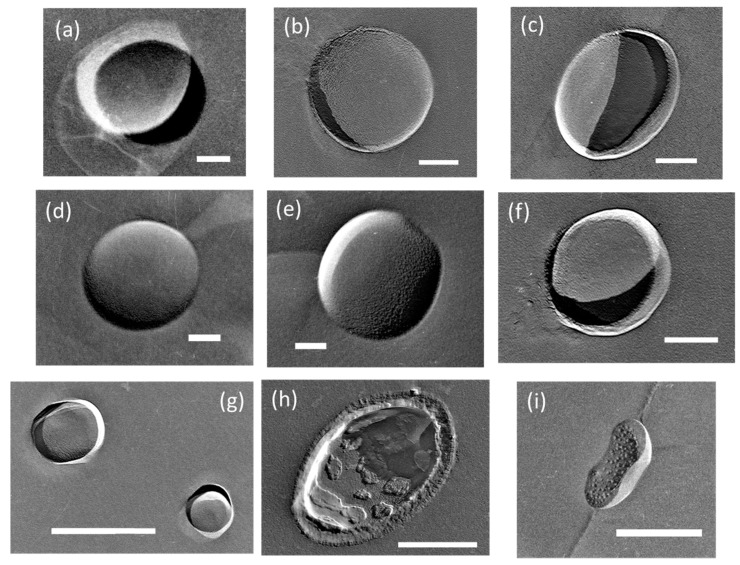
Typical TEM images of UFBs in each solution. (**a**) **I-W**, (**b**) **I-BT**, and (**c**) **II-BT** as initial conditions, (**d**) **I-P** and (**e**) **II-P** as transporting conditions, (**f**) **I-R** and (**g**) **II-R** as near-plant conditions. Each symbol shows the following: I = prepared with UFB generator, II = control water without UFB generator. The other symbols indicate location: BT = buffer tank; P = irrigation pipe; and R = rhizosphere. **I-W** is the tap water before entering the UFB generator. (**h**) A unique UFB from **I-R** solution that had accumulated much impurity. (**i**) Impurity masses observed independently of UFBs in **II-R** solution. Each scale shows 100 nm except for those in (**g**–**i**), which show 500 nm.

Consider the UFB replicas. In the replicas, we found spherical or elliptical holes with a diameter of several hundred nm in every replica film. For example, Figure 1 shows TEM images of typical UFBs observed in seven solution samples, including samples from the UFB-added solution (I) and the control solution without added UFBs (II) (see Figure 8 for sample locations). The samples from the buffer tank are called **I-BT** and **II-BT** as initial conditions, from the irrigation pipe are **I-P** and **II-P** as transporting conditions, and from the puddle near the root are **I-R** and **II-R** as near-plant conditions. The tap water sample used for the UFB solution preparation is called **I-W**, which is also referred to as the initial condition. We found that enough UFBs, at least 10^6^ mL^−1^ in number concentration, were present in each solution sample to determine the size distributions with the freeze-fracture replica method.

Except for the pure-water case, the images show a thin layer, about 10-nm thick for most samples, on the UFB surface (e.g., Figure 1b). This layer closely resembles that observed on UFBs dispersed in wastewater (Figure 5 in [6]) or that observed in NaCl solution (Figure 8 in [7]). Thus, we consider that this thin layer is a concentrated layer of impurities, likely coming from solutes in the nutrient solution.

The thickness of this “impurity layer” was often much greater in the rhizosphere **I-R** and **II-R** samples (e.g., Figure 1f). In such a UFB, impurity masses are sometimes found clustered into islands on the surface (Figure 1h). Moreover, Figure 1i shows that impurity masses can also occur away from UFBs. Regarding the distinction between these impurity masses and UFBs, we examined the roughness (concave or convex) of the objects using the direction of shadow when platinum was deposited. As the “impurity layers” and impurity deposits are thicker below the peat moss (i.e., in the **I-R** samples), we consider that the thick layer as seen in Figure 1h likely formed when the UFBs passed through the soil.

### 3.2. Size Distribution of UFBs

To obtain quantitative data on UFBs in the solution, we prepared five replica samples from each type of solution and each sample location. Then, for each sample, we examined the bubble forms in at least five areas with the TEM. Assuming that the bubble shapes are elliptical, we measured the long and short diameters in the images, and then calculated the equivalent diameter *d* of the circle having the same cross-sectional area. These diameters were put into 1 of 13 size classes (100 nm bin-size). As the number of observed UFBs is different in each area of a given sample, the number in each size class is normalized by the total observed number *n* of UFBs in all five (or more) areas of the sample (*n* is about 100). We then determined the total number density *N* in the sample solution (estimated from *n*, described later), and multiplied the above fraction for each size class to determine their number density. Then, we obtained the diameter histogram and calculated the average bubble diameter <*d*>.

**Figure 2:**
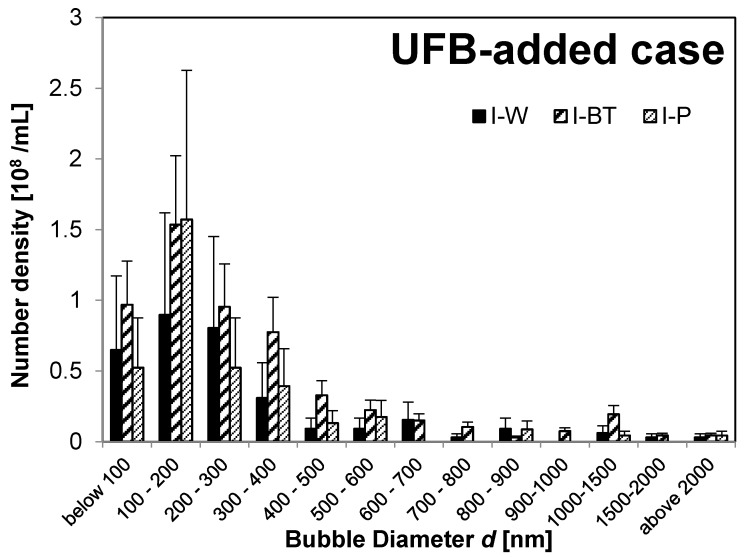
Number of fine bubbles of UFB-added case: **I-W** (solid black), **I-BT** (thick oblique line), and **I-P** (thin oblique line).

**Figure 3:**
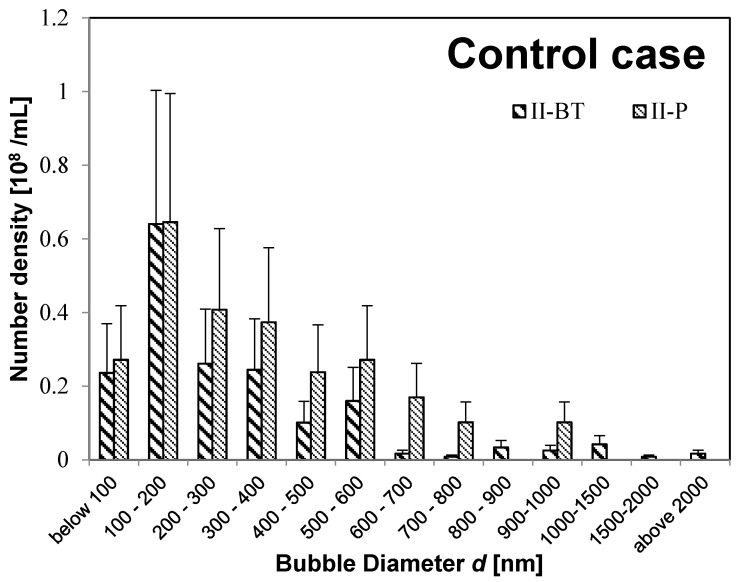
Number of fine bubbles of control case: **II-BT** (thick oblique line), and **II-P** (thin oblique line).

First, we consider the size distribution and state of the UFBs in nutrient solution before feeding. We find that the UFB distributions peak at diameters *d* below 300 nm in all solutions, but some MBs have *d* > 1 μm. For example, Figure 2 shows the bubble-diameter histograms of the UFB-added case solutions **(I-W** solution, **I-BT** solution, and **I-P** solution), whereas Figure 3 shows the results of the control case (**II-BT** solution and **II-P** solution).

The **I-W** and **I-BT** solutions have nearly the same UFB size distributions, meaning that the addition of the nutrient solution (about 0.3%) did not change the size distribution. However, the TEM images in Figure 1b, and d show that an impurity layer forms on the surface of UFBs in the nutrient solution. So, if the amount of nutrient solution were to increase, the size distribution of UFBs may begin to be affected by the accumulation of impurities on the UFBs.

Moreover, the buffer-tank UFB-distribution is nearly the same as that for the irrigation pipe for both the UFB-added case (**I-BT** and **I-P** in Figure 2) and the control case (**II-BT** and **II-P** in Figure 3). Therefore, the UFB-distribution in the nutrient solution in the buffer tank was supplied to the plant without change in the UFB-distribution. However, the UFB-supplied water (**I-P**) has significantly more fine bubbles compared to that in the control zone (**II-P**).

Although the control solution had no added bubbles, it nevertheless had UFBs after the freeze-fracture observations. Since the minimum number density of UFBs for the freeze-fracture replica method is the order 10^6^/mL, the observed UFBs in control cases are significant. We will discuss the possible origin of these UFBs in Section 3.4.

**Figure 4:**
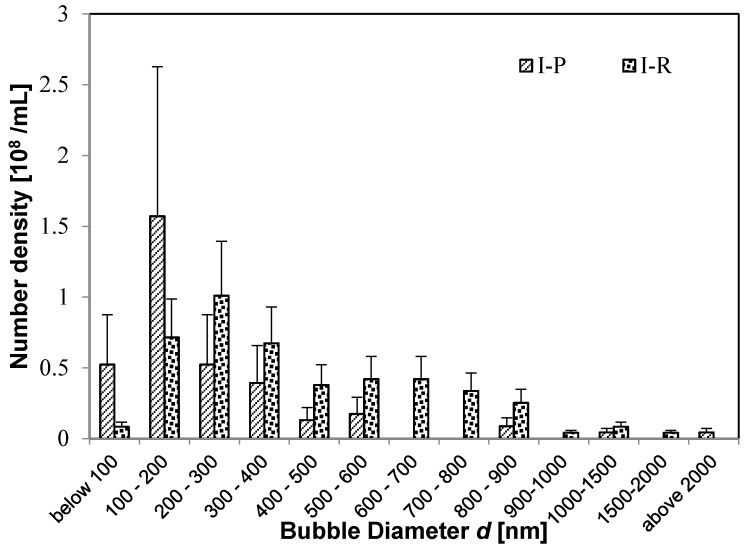
Number of fine bubbles in the UFB-added case before and after reaching plant roots. Before: **I-P** (thin oblique line). After: **I-R** (thick points).

**Figure 5:**
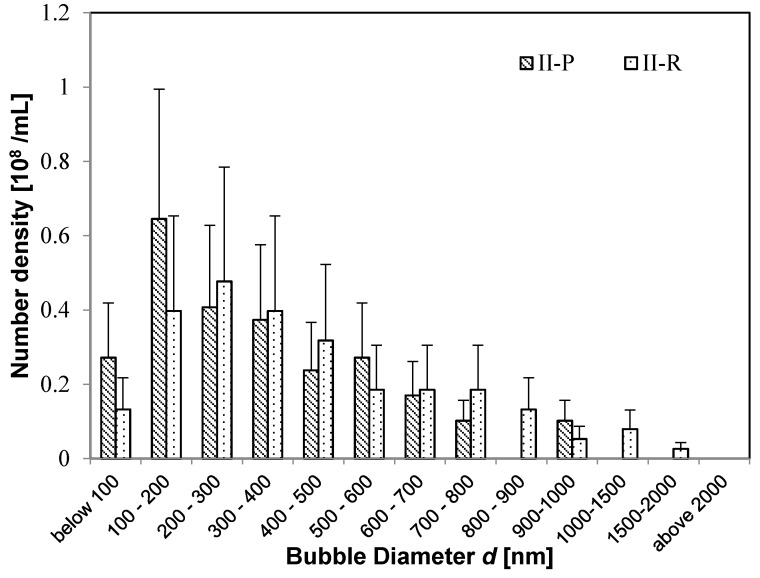
Same as Figure 4 except for the control case. Before supply to plants: **II-P** (thin oblique line). After reaching roots: **II-R** (thin points).

Next, we examine how the distribution of UFBs change after reaching the plants. For the UFB-added case, Figure 4 shows that the size distribution of UFBs shifts to larger sizes upon reaching the rhizosphere. The same shift occurs for the control case (Figure 5). Thus, almost the same number of UFBs remain in the solution even after the solution passes through the soil layer. Figure 1 shows that the surface of UFBs in **I-R** and **II-R** solutions tends to have impurity. Thus, we speculate that the UFB size will increase as small UFBs either coalesce or undergo the Ostwald-ripening process.

When the size distributions of **I-R** solution and **II-R** solution are compared, they are very similar even though their distributions differ in the irrigation pipe (Figure 4 and Figure 5). This result shows that UFBs tend to stabilize upon reaching several hundred nm, and that the size distribution of UFBs tends to a more nearly uniform distribution. It is interesting that this size distribution resembles the UFB distribution in a 100-mM NaCl solution, from a study in which the UFBs were more stable when a solution contained a small amount of NaCl ([7] Figure 3b).

### 3.3. UFB Number Concentration and Average Diameter

**Figure 6:**
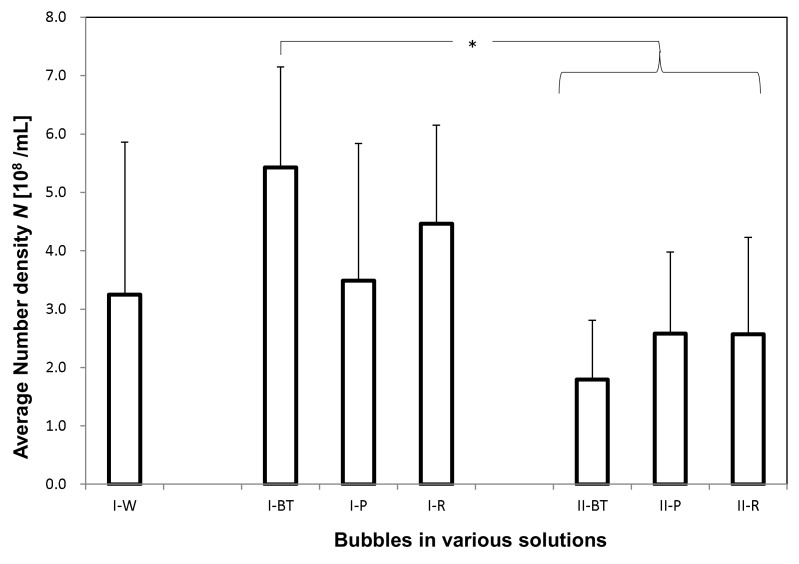
Total fine-bubble number concentration *N* of each solution. Each error bar shows the standard deviation of measurements. The “*” indicates that a significant difference was observed with the Tukey–Kramer test (Microsoft Excel 2010 and Bell Curve for Excel; *p* < 0.05).

**Figure 7:**
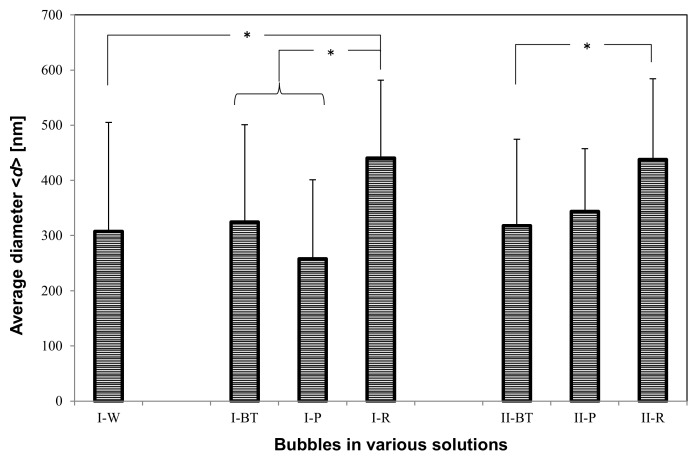
Same as Figure 8 except for mean diameter <*d*> of fine bubbles in each solution.

The total number concentration *N* (Figure 6) and the arithmetic-averaged UFB diameter <*d*> (Figure 7) were determined by averaging all UFBs observed in each solution sample. For the **I-W** solution, *N* = 3.3 ± 2.6 × 10^8^/mL and <*d*> = 3.1 ± 2.0 × 10^2^ nm, which roughly agrees with the set value of the UFB generator. On the other hand, the value of *N* in the **I-BT** solution is 5.4 ± 1.7 × 10^8^/mL, slightly larger but not significantly different from that in the **I-W** solution (Figure 6). The measurement uncertainty (standard deviation: SD) of the **I-W** solution is particularly large due to the observed large concentration variations of the UFBs. Concerning the diameters, their values also were consistent, with <*d*> = 3.2 ± 1.8 × 10^2^ nm in the **I-BT** solution (Figure 7). As the histograms of the solutions are also similar (see Figure 2), it appears that the addition of the nutrients does not significantly affect the stability of the UFBs.

Figure 6 and Figure 7 also show that both *N* and <*d*> in **I-BT** solution and **I-P** solution are consistent within their uncertainties. Thus, it appears that the supply line from the buffer tank preserves the UFBs. We find the same conclusion holds for the control case.

However, the value of <*d*> in the solutions slightly increases between the buffer tank and the plant roots (Figure 7). This reflects the results observed in the histograms (Figure 4 and Figure 5), in which the number of fine UFBs decreased while the number of submicron bubbles increased. Yet the total value *N* (Figure 6) shows no significant change. This finding indicates that transport through the peat moss does not significantly affect the number of UFBs in the UFB-added solution. In addition, the increase in size (together with the TEM images showing a thicker UFB–water interface) suggest that the passage through the soil causes impurities to accumulate on the UFB surface, increasing the UFB size.

Comparing the control and UFB-added cases, the average UFB diameters <*d*> are nearly the same (Figure 7), whereas the value of *N* is significantly smaller in each location for the control case (Figure 6). That is, the solutions in the UFB-added case contain significantly more UFBs due to the artificially generated UFBs.

We conclude that the number of UFBs observed in the UFB-added solutions roughly equals the set level of the UFB generator that is being manufactured at the plant factory, in which *N* is about 4 × 10^8^/mL and <*d*> is about 300 nm. The number of UFBs is roughly conserved through to the rhizosphere, but the average diameter increases about 50%. Although there were also UFBs in the solutions of the control zone, they were likely generated mostly during the nutrient solution preparation and freeze-fracture replication. The reproducibility of these results was confirmed by repeating these measurements at different times (June 2015 and October 2016).

### 3.4. Source of UFBs Observed in Control-Case Solutions

We now consider why UFBs are observed in the control-case solution. Two possible sources of UFBs are: (1) being formed during replica preparation as ‘secondary bubbles’ and (2) having existed in the solution as ‘primary bubbles’.

Secondary bubbles can form in the solution from dissolved air at the freezing front because the solubility of air in ice is negligible compared to that in water. For example, Lipp et al. [12] studied secondary bubble formation under different freezing speeds and found that the bubbles were smaller and greater in number under a higher freezing speed.

To estimate the amount of secondary bubbles, we first estimate the amount of air in the water that will form bubbles during freezing. This amount depends on the freezing speed *V_f_* and the diffusion coefficient of air in water D_air_. In our study, the cooling rate of the solution is about 10^3^ K/min [7], which corresponds to a freezing speed *V_f_* of about 10^5^ μm/s. From this value, the diffusion boundary layer in front of the ice-water interface is calculated as D_air_/*V_f_*. Not knowing the precise D_air_ value at freezing temperature, we assume it equals that of oxygen in water at atmospheric pressure and room temperature, which is 2 × 10^−9^ m^2^/s [13]. The resulting thickness D_air_/*V_f_* is about 10^−8^ m. Within this region, we consider that the air molecules cannot diffuse away during freezing, meaning that they instead collect into bubbles that are frozen immediately in ice.

Now consider how many secondary bubbles can be formed from the dissolved air. The amount of air molecules in pure water is assumed to be about 0.8 times that in saturated conditions. This is caused by the ratio of the dissolved oxygen (DO) value between that in pure water (5.5) and that in UFB-added water (6.9), in which the dissolved gas is considered to be saturated [7]. If all dissolved air converted to UFBs having its diameter <*d*> = 300 nm (about the average diameter of UFBs in the control-case solution, in Figure 7) and its inner pressure ∆*P* is calculated from Equation (2), the number concentration of the secondary bubbles N* would be

N* = 0.8s RT/(P_0_ + ∆P) V_bub_ v_N_, (3)

where *s* = 0.019 cm^3^/mL [14] is the solubility of air in water at 293.2 K, R is the molar gas constant, *T* is temperature, *P*_0_ is atmospheric pressure, *V_bub_* is the volume of a UFB with <*d*> = 300 nm, and *v_N_* is the volume of 1 mol of a gas at standard temperature and pressure. The resulting N* is about 1.1 × 10^11^/mL. Uchida et al. [7] observed UFBs of order 10^7^/mL from the replica of pure water at room temperature by the same freeze-fracture replica method. If these UFBs were secondary bubbles, this means that the nucleation probability of a secondary bubble in pure water is about 10^−4^. However, the nutrient solution used in the present study included various solutes that would concentrate at the ice-water interface during freezing. Such a concentration of solute would increase the nucleation probability. A ten-fold increase would explain the number of UFBs in the control case here. This estimation contains several assumptions about the freezing process, bubble formation, and physical parameters, and thus should be considered as a very rough estimate. However, it suggests a plausible explanation for the observed UFBs.

Some of these UFBs may instead be primary bubbles. One possible source of these additional UFBs is a size-reduction of MBs and/or milli-bubbles produced during the nutrient solution preparation process. For example, when comparing the size distribution of UFBs before being supplied to the plant body (Figure 2 and Figure 3), the **II-P** solution has more UFBs above 500-nm diameter than the **I-P** solution. Figure 3 also shows that, when compared in the buffer tank and in the irrigation pipe in the control zone, the number of MBs of 1 μm or more decreases while the UFBs of submicron order increases. In the replica observation method, most MBs larger than 5 μm are difficult to measure because they are usually located at the edge of the observed area. So, the distribution of fine bubbles larger than 5 μm would be slightly underestimated. To evaluate this possibility quantitatively, we should know the whole range of fine bubbles, ranging from 10^−4^ to 10^−8^ m. However, we do not yet have a technique to measure such a wide range. For example, the freeze-fracture replica method covers diameters of 10^−6^ to 10^−8^ m, whereas the particle-tracking analysis method covers only 10^−7^ to 10^−8^ m [15].

### 3.5. Possible Plant-Growth Promotion Mechanisms by UFBs in Nutrient Solution

UFBs are thought to promote the growth of plants [1,2]. However, its mechanism has not been determined yet. One proposed mechanism is in the germination process. Liu et al. [4] revealed that the presence of UFBs induced a constant development of radicals in nutrient solutions, thereby promoting germination. However, the mechanism of promoting the growth of growing plants and the magnitude of their effect are still under investigation.

The mechanism may involve either the UFB air content or the interface. Due to the air/water interfacial tension, the pressure inside a UFB should be relatively high according to Equation (2). However, various substances readily accumulate at the interface [7], as shown by the impurity deposits in Figure 1, and such a change at the interface should decrease the air/water interfacial tension. As a result, the actual internal pressure would be lower than that predicted by use of the air/water interfacial tension of bulk water [16]. Therefore, the bubble internal pressure in an actual solution should be smaller than that estimated from Equation (2). We also consider that the UFB may be stabilized by a diffusion barrier [17].

Takahashi [9] pointed out one of the main mechanisms by which UFBs act lies in their action as a gas buffer in the liquid phase. As UFBs provide a large gas-liquid interface in the liquid and their internal pressure is high, gas is constantly supplied from UFBs into the nutrient solution for as long as UFBs are present. Thus, the nutrient solution can be considered to maintain a saturation state of the gas. Uchida et al. [7] measured the time change of the dissolved oxygen (DO) value of the O_2_-UFB-added solution and confirmed that the aqueous solution was in the O_2_ supersaturated state during the UFB-existence period. In the present study, the DO value of each nutrient solution was measured to be a constant value of 6.4 mg/L (at 298 K) in the solution from the buffer tank to just before reaching the plant. In other words, due to the UFBs, the nutrient solution in this plant factory is expected to be supersaturated in O_2_, and thus O_2_ should be efficiently supplied to the plant body.

Consider now the interface. The surface of a UFB is negatively charged, and thus cations in the nutrient solution might concentrate at the UFB interface. A concentration of solutes (such as calcium, magnesium, potassium, and ammonia) may explain the thin membrane-like structure that appears on the UFB in the nutrient solution supplied to the plant body. Moreover, as the UFB-added solution passes through the peat moss, the peat moss should add other impurities to the solution that may deposit on the surface of the UFBs (Figure 1h). Therefore, it is possible that the UFBs help transport concentrated nutrients to the rhizosphere, and then into the plant body. As the surface of the UFB is also a gas-liquid interface, the UFBs also may help transport hydrophobic materials and solid solutes.

On the other hand, the effect of UFBs on growth is unlikely to come from pH. The pH of the nutrient solution was approximately constant at about 5.8 within the range from the buffer tank to the irrigation pipe. This pH value is also about 5.8 in the control nutrient solution, so if a growth-promotion effect is verified, then it cannot be due to a difference in pH value. Also as the influence of pH on the ζ potential value is small if pH is around 5.8 [7,9], we assume that there is no influence of pH on the UFB distribution.

Therefore, the results of this research indicate that UFBs continue to exist stably even while moving through a supply pipe from a buffer tank and through peat moss. Through their transport, UFBs supply gas molecules into the solution and collect impurity on their surface, some of which may benefit the plant. However, as all the observations were on replicas, we could not identify what substances were on the UFB surface. Also, we still need to clarify how UFBs may promote plant growth.

## 4. Materials and Methods

**Figure 8:**
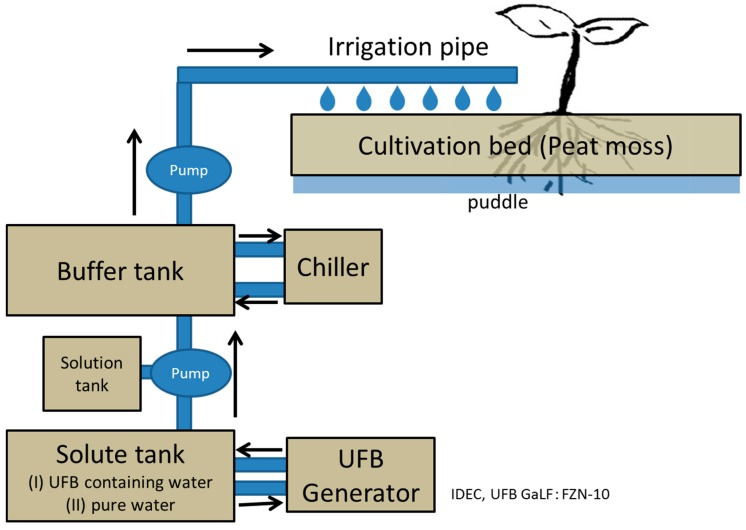
The plant factory with ultra-fine bubbles (UFB)-added medium supply system. A second line system, identical except with pure water as a solute without UFB generator, is used for the control case.

In a plant factory of the JNC Corporation (Minamata Research Center), 552 stems of midi tomatoes are grown at temperature of about 298 K. The nutrient solution for cultivation is prepared by circulating 300 L of tap water at the rate of 50 L/h for 5 to 6 h at room temperature to a fine-bubble-producing apparatus (IDEC UFB GaLF: type FZN-10), such that the generated UFB concentrations reaches 4 × 10^8^/mL. Then, this air–UFB-added water, hereafter just ‘**UFB water**’, is mixed with 8.7 L of the nutrient solution in a buffer tank. The result is UFB-added nutrient solution (called ‘**UFB-added case**’), labeled I. On the other hand, as a control, ordinary nutrient solution (referred to as ‘**control** **case**’) and labeled II was prepared by using 100 L of tap water instead of the UFB water. The UFB-added nutrient solution (I) and the ordinary nutrient solution (II) are pumped from a buffer tank (adjusted to around 298 K) through the irrigation pipe, to the midi tomatoes cultured on the peat moss (Figure 8). Both the dissolved oxygen (DO) value and pH value of the nutrient solution were also measured in the buffer tanks and in the irrigation pipes by handy-type meters (Custom: type DO-5509 and Takemura Denki Seisakusho: type PCT35, respectively).

For a replica sample, about 10–20 mm^3^ of solution is extracted and then immediately quenched in liquid nitrogen. For both the (I) UFB-added case and (II) control case, the sample locations are in the buffer tank **BT**, the end of the irrigation pipe **P**, and near the roots at the bottom of the incubator on a vinyl mat **R**. The UFB water is also sampled (**I-W** solution). All quenched samples were transported to Sapporo at liquid nitrogen temperature, and used for preparing replicas as mentioned below.

We just briefly describe the sample-preparation for the freeze-fracture replicas and TEM observations here as the details are described in [6,7,16]. Each frozen sample (at liquid nitrogen temperature) is placed in a freeze-etching device (JEOL, Tokyo, Japan, type JFD-9010), and fractured by a knife edge in a vacuum chamber at a temperature of about 150 K and a pressure of about 10^−5^ Pa. The form of bubbles or aggregation of solutes appearing in the fractured ice cleavage section is then copied as a replica by depositing platinum and carbon from an oblique direction on the section. The ice sample with the replica membrane is then taken out of the apparatus and transferred to a TEM-observation copper-grid (Nisshin EM, Tokyo, Japan, type F-400; opening size 43-μm square) by melting the ice body. The replica is observed with TEM (JEOL, type JEM-2010), and the form of UFB recorded on an imaging plate (FDL-UR-V, manufactured by Fuji Photo Film, Tokyo, Japan). Then, the number and size of bubbles observed in an opening of the grid are measured, and a size histogram and the number density of UFBs are estimated by assuming a uniform distribution of UFBs. Uncertainties of the measured values are evaluated by taking into account the variations on one replica (more than five grid openings were observed for measuring UFBs in a given replica sample) and the variations among multiple replica samples (five frozen samples were prepared from each extracted solution).

## 5. Conclusions

We used TEM observations on freeze-fracture replicas to measure distributions of UFBs in the UFB-added nutrient solution for a plant factory. In the nutrient solution, UFBs of about 300 nm in diameter were found to exist at an average number density of 4 × 10^8^/mL. UFBs were also measured in the control solution, a solution made with tap water without the UFB generator, and found to have about half the total number of UFBs.

With the added nutrient solution, the total number of UFBs was conserved through the supply line to the vicinity of the root. In addition, a layer of accumulated impurities formed on the UFB surface and thickened after passing through the peat moss. During this supply process, the number of UFBs of diameter 200 nm or less slightly decreased, while those with diameters of about 300 nm to 1 μm increased. Overall, the average diameter increased about 1.5 times. This finding suggests that the UFB properties that change, such as the accumulation of impurities on the surface and their size distributions, should be considered when examining how UFBs affect the plant body.
